# Prevalence of sleep-related symptoms in patients receiving methadone maintenance treatment: a systematic review and meta-analysis

**DOI:** 10.1186/s13722-026-00690-2

**Published:** 2026-06-19

**Authors:** Domitille Palix, Benjamin Petit, Benoit Trojak, Anastasia Demina

**Affiliations:** 1 Université Bourgogne Europe, CHU Dijon Bourgogne, Dijon, France; 2 INSERM U1093, CAPS, Université de Bourgogne, UFR STAPS, Dijon, France

**Keywords:** Opioid maintenance treatment, Methadone, Sleep disorders, Prevalence

## Abstract

**Background:**

Sleep disturbances are sometimes reported in clinical settings among individuals with opioid use disorder (OUD) receiving methadone maintenance treatment (MMT). Although numerous studies have explored this issue, a systematic assessment of the prevalence of these disturbances is lacking.

**Methods:**

We searched for trials reporting the prevalence of sleep disturbances in patients with OUD receiving MMT. Studies assessing sleep-related symptoms using a standardized scale were included in a prevalence meta-analysis. All other studies were synthesized narratively and in tabular form.

**Results:**

Thirty-eight articles were included. Thirteen studies assessed the prevalence of poor sleep quality using a Pittsburgh Sleep Quality Index (PSQI) score > 5. Given the high level of heterogeneity among included studies, a prediction interval was calculated, suggesting that the prevalence of poor sleepers in future trials will likely range from 50% to 93%. The weighted prevalence estimate of poor sleepers was 75% (95% CI [61–85%], τ² = 1.34). Sensitivity analyses were performed to investigate heterogeneity among the included studies. Eight of the thirteen studies were rated as having low risk of bias, and the certainty of evidence was found to be moderate. In the narrative synthesis, the reported prevalence of daytime sleepiness ranged from 15% to 50%. In studies using polysomnography or nocturnal polygraphy, patients exhibited significantly reduced sleep efficiency and a high prevalence of sleep apnea syndrome, ranging from 39.1% to 78.3%.

**Conclusions:**

Our findings indicate that patients receiving MMT often experience poor sleep quality, highlighting the need for systematic attention to sleep issues while taking into account psychosocial vulnerability.

**Registration:**

CRD42025649687.

**Clinical trial number:**

Not applicable.

**Supplementary Information:**

The online version contains supplementary material available at https://doi.org/10.1186/s13722-026-00690-2.

## Introduction

Methadone, a synthetic opioid, is a pure agonist of morphine mu receptors [1]. Methadone maintenance treatment (MMT) is one of the medications currently indicated for treating opioid use disorder (OUD) and is a standard of care for many patients with OUD [2, 3]. Unlike buprenorphine, another medication for OUD, methadone does not have a ceiling effect. This can lead to overdoses when the dosage surpasses the patient’s tolerance or when used concurrently with other central nervous system depressors such as alcohol, benzodiazepines or heroin [3]. Initially, the patient receives a low dose of methadone which is then gradually increased in combination with regular clinical observations on tolerance and efficacy in order to alleviate withdrawal and to decrease craving. After this closely supervised inpatient or outpatient induction, the outpatient maintenance phase takes place. Given methadone’s pharmacological profile and the associated risks, methadone prescription is limited to certain professionals and is generally implemented in specialized facilities [3]. MMT has repeatedly demonstrated beneficial effects on morbidity and mortality in patients with OUD and its cost-effectiveness has been proven [1, 2, 4, 5]. MMT has been shown to influence a wide range of health-related parameters, including improvement in OUD outcomes such as withdrawal symptoms and craving reduction, recovery facilitation, and decreased risk of relapse.

During the initiation of MMT and the maintenance stage, patients with OUD sometimes express sleep-related complaints [6, 7]. Sleep disturbances are a common problem among people with OUD. The sedative properties of opioids lead to daytime sleepiness and disruptions in sleep architecture and circadian rhythm [8]. Therefore, sleep disturbances are often present before treatment is started and are generally exacerbated during withdrawal. While MMT can help stabilize some aspects of these disruptions, it can also contribute to poor sleep [9].

Poor sleep quality is known to increase the risk of developing chronic diseases such as type 2 diabetes, hypertension, and psychiatric illnesses such as depression. In patients receiving MMT, poor sleep quality can also hinder the treatment and rehabilitation process and represent a risk factor for relapse. It is therefore of the essence to understand this phenomenon, particularly in this at-risk population due to their generally poorer health and reduced access to health care compared with the general population [3].

Many previous studies have sought to understand and characterize sleep disturbances associated with MMT and to propose informed and evidence-based solutions, but a systematic assessment of the prevalence of these symptoms is lacking. We aimed to apply this systematic approach to the existing literature and to discuss the findings from a clinical perspective. All authors declare no competing interests relevant to the subject of the manuscript.

## Materials and methods

### Search strategy

Our systematic review protocol was registered on the PROSPERO platform with the registration number CRD42025649687 [10].

We searched articles from inception to October 2024 using four electronic databases (CENTRAL, PubMed, Embase, and PsycINFO) for published trials and three clinical trials registries (ClinicalTrials.gov, EU Clinical Trials, WHO Clinical Trials Registry Platform) for unpublished or ongoing studies. The search equation was created by DP and BP and adapted for all databases and registries. It combined index terms and text words for various sleep-related problems and methadone. All adapted strategies are presented in the Supporting information (Document S1).

All search hits were added into a reference manager (Zotero), and then into systematic review software (Rayyan) [11, 12]. Semi-automatic duplicate removal was performed, with the identified duplicates systematically confirmed by DP and AD.

### Study selection

Study selection was carried out independently and in duplicate by two authors (DP and AD) using Rayyan’s blind mode. The first round of selection was performed based on titles and abstracts, and conflicts were adjudicated by BP. The remaining trials were screened based on full texts by DP and AD using the same procedure.

We included articles published in English or French without time or geographical limits. We included studies in humans with diagnosed OUD receiving MMT treatment. Studies in which methadone was given for pain management or as part of opioid withdrawal management without the maintenance component were excluded. We included randomized controlled studies (RCTs), non-randomized studies, cross-sectional observations, and cohort studies. Studies were eligible for inclusion if they reported both the total number of included patients and the number of patients with sleep-related problems, thereby allowing prevalence to be calculated. Therefore, studies including only patients with sleep-related problems, case reports and case series were excluded. Sleep-related complaints or problems were considered broadly as sleep-related symptoms and were not limited to formally diagnosed sleep disorders. Studies were eligible if they reported sleep-related symptoms either as part of a systematic assessment using validated scales or clinical interviews, or as adverse events among patients receiving MMT. This approach was chosen to maximize data collection and to examine whether prevalence estimates differed between studies that systematically assessed sleep-related symptoms and those that reported spontaneous complaints or adverse events.

### Extraction

Data on study and patient characteristics, methadone dose and duration, and the prevalence of sleep-related symptoms were extracted using an extraction form by two authors (DP and AD) independently and in duplicate. Discrepancies were settled with a third author (BP). Prior to data extraction, the extraction form was pilot tested on three trials by DP and AD. No adjustments were needed after pilot testing. The extraction form with all extracted information is presented in Table 1.

**Table 1:** Characteristics of the included studies

*N*	STUDY ID	DESIGN	SAMPLE SIZE	POPULATION	METHADONE REGIMEN	SLEEP DISORDER EVALUATION AND RESULT
1	Langrod, 1981, USA	Observational: cross-sectional evaluation	*N* = 52 for long term patients (> 2 years in MMT) and *N* = 49 for new patients (< 5 months in MMT)	Female: 0% Age: no information	Median methadone dose (mg/day): for long term patients, 100 IQR (80; 130), for new patients 100 IQR (65; 110). Mean duration in MMT (months): for long term patients, 30.8 (24 to 42), for new patients, 3.5 (1.5 to 5)	Survey exploring physical complaints: 23% of new patients and 27% of long-term patients were bothered by sleepiness at time of interview.
2	Teichtahl, 2001, Australia	Observational: cross-sectional evaluation	*N* = 10	Female: 40% Age: 33 (6) years	MMT dose: between 50 and 120 mg/day	Polysomnography: 60% of the patients had apnea-hypopnea index (AHI) > 5, 50% of the patients had AHI > 10. 60% of the patients had a central apnoea index > 5 and 40% had a central apnoea index > 10. Patients with MMT had a significantly lower sleep efficiency.
3	Mattick, 2002, Australia	Randomized clinical trial, only participants from methadone group considered	*N* = 205	Female: 31% Age 30 (8) years	Induction then Mean MMT dose at week 13(mg/day): 57.3 (range 10–150)	Adverse events report: Insomnia was reported in 10% and somnolence in 9% of the participants.
4	Stein, 2004, USA	Observational: cross-sectional evaluation	*N* = 225	Female: 45.9% Age 40.68 (7.68) years	Methadone dose > 100 mg in 102 (39.7%) individuals	Self-report scales: PSQI: 83.9% of the participants reported PSQI > 5 There was an association between depression, anxiety, nicotine dependence, pain, and unemployment and poorer sleep quality.
5	Lofwall, 2005, USA	Randomized clinical trial, only participants from methadone group considered	*N* = 80	Female: 26.2% Age: 32.7 (6.0) years	Mean MMT dose (mg/day): 54 (range 50–90)	Adverse events report: Poor sleep was reported in 1.25% of the participants
6	Peles, 2005, Israel	Observational: cross-sectional evaluation	*N* = 101	Female: 21.8% Age: 40.4 (9.6) years Psychiatric comorbidity: 52.5% Chronic pain: 46.5% Benzodiazepine use: 46.5%	Mean MMT dose (mg/day): 157.0 (52.9)	Self-report scale: PSQI: 75.2% of patients reported PSQI > 5. PSQI was higher in participants who received benzodiazepines, reported chronic pain and psychiatric comorbidity. There was a correlation of PSQI scores with years of opioid abuse and methadone dose.
7	Wang, 2008, Australia	Observational: cross-sectional evaluation	*N* = 50	Female: 25 (50%) Age: 35 ± 9 years	Participants were in an MMT program for at least 2 months and receiving a stable dose of methadone	Polysomnography Central apnea index (CAI) and obstructive sleep apnea-hypopnea index (OSAHI) 15 participants (30%) had CAI > 5 and 10 (20%) > 10. Participants had significantly less REM sleep and stage 1, and more stage 2 sleep. Methadone blood concentration was significantly associated with the severity of CSA. Self-report questionnaire: ESS: 12 participants (24%) had ESS > 10 Functional Outcome of Sleep Questionnaire (FOSQ): 70% of patients receiving MMT had a total FOSQ < 17.9
8	Wolstein, 2009, Germany	Randomized clinical trial, only run-in phase data extracted (4 weeks post-inclusion)	*N* = 93	Female: 38.7% Age: 29.6 (6.2) years	Mean MMT dose (mg/day): 67 (26) and 70 (25)	Adverse events report: Sleep disorders were seen in 20% of all patients receiving methadone
9	Hsu, 2011, Taiwan	Observational: cross-sectional evaluation	*N* = 121	Female: 13.2% Age: 39.6 (8.6) years Regular hypnotic treatment: 9.1% Alcohol use: 23.1%	Mean MMT dose (mg/day): 51.4 (24.9)	Self-report scales: PSQI: 70.2% of patients had PSQI scores > 5 There was a correlation between PSQI and methadone dose.
10	Lee, 2011 (data from an abstract issue)	Observational: cross-sectional evaluation	*N* = 82	No information	No information	Self-report scales: PSQI: 67.9% of patients receiving MMT reported poor sleep quality ESS: 25.6% of patients receiving MMT reported excessive daytime sleepiness
11	Peles, 2011, Israel	Observational: prospective longitudinal study	*N* = 23	Female: 17.4% Age: 43.9 (11.3) Psychiatric comorbidities: 69.6% HCV: 56.5% HBV: 13.0% HIV: 17.4% Opioid use: 100% Cocaine use: 43.5% Amphetamine use: 8.7% Benzodiazepine use: 78.3% Chronic pain: 65.2%	Participants were followed 6 and 12 months after initiation of MMT. Mean MMT dose (mg/day) at 6 months: 133.5 (31.1), at 12 months: 141.1 (33.5)	Self-report scale Mean (SD) PSQI at baseline, 6 months and 12 months: 11.2 (4.9), 11.0 (5.6) and 10.9 (4.0) Polysomnography: 26.1% of the participants reported clinical obstructive apneas at baseline, and 39.1% and 43.5% reported clinical obstructive apneas at the 6- and 12-month follow-up.
12	Wang, 2012, Taiwan	Observational: cross-sectional evaluation	*N* = 366	Female: 18.9% Age 38 years	Mean methadone dose (mg/day): 54.7 (28.1) Mean MMT duration (weeks): 64.7 (39.1)	Assessment of the adverse events by research nurses using Treatment Emergent Symptoms Scale (TESS). Symptoms were counted as related to methadone treatment only if they occurred after the start of MMT. The severity of the symptom was evaluated using a 3-point Likert scale (mild, moderate and severe). Insomnia was reported by 18.3% of the participants.
13	Nkire, 2013, Ireland	Observational: cross-sectional evaluation	*N* = 89	Female: 24% Age: 40 years	No information	Self-report questionnaire designed by the authors: 65% of respondents reported sleep difficulties (of these, 100% - initial insomnia, 93% - middle insomnia, and 66% - terminal insomnia).
14	Cernovsky, 2014, Canada	Observational: cross-sectional evaluation	*N* = 56	Female: 39.3% Age: 34 (9) years	Mean MMT dose (mg/day): 48.7 (25)	Rating sleep disturbance over 7 days using a 0 to 4 Likert scale for 3 symptoms: difficulty falling asleep, early morning awakening and disturbed sleep. 62.5% reported problems falling asleep 53.6% reported early morning awakenings 64.3% reported restless or disturbed sleep
15	Hämmig, 2014, Switzerland	Randomized clinical trial, only cross-over phase data extracted (22 weeks post-inclusion)	*N* = 260	Female: 18.5% Age: 38.1 (7.6) years	Mean methadone dose (mg/day): 105.51 (39.59)	Adverse events report: Sleep disorders were seen in 4.1% of all patients receiving methadone
16	Peles, 2014 Israel	Observational: cross-sectional evaluation of sleep quality in patients receiving MMT with take-home doses.	*N* = 123	Female: 34 (27.6%) Age: 47.2 (10.5) years ADHD in 51 (41.2%) patients	Mean duration in MMT treatment was 7.6 (5.5) years Mean methadone dose (mg/day): from 106.9 ± 48 to 126.8 ± 39	Self-report scales: PSQI: Mean 8.2 (4.1), > 5 for 86 (69.9%) participants ESS: Mean 7.6 (3.8), > 6 for 59 (48%) participants Patients with higher number of take-home dosages of methadone had better sleep quality, less daily sleepiness and less cognitive impairment.
17	Enguelberg-Gabbay, 2016, Israel	Observational: cross-sectional evaluation	*N* = 69	Female: 0% Age: 32.4 (9.7) years All participants were incarcerated	Participants were maintained on methadone treatment, following a standard protocol	Diagnosis of sleep bruxism was based on the American Academy of Sleep Medicine criteria 52.2% of the participants were diagnosed with sleep bruxism.
18	Nordmann, 2016 France	Multisite, open label, randomized and controlled, non-inferiority trial	*N* = 173	Female: 16% Age: 33 years (range = [20–57]; IQR = [27–38]) 119 individuals used cocaine, anxiolytic or hypnotic substances, 15% reported injection in the past month. 13% had AUDIT score indicating possible alcohol use disorder.	Data for sleep disturbances are extracted for baseline, 6 and 12 months after methadone initiation.	Center for Epidemiological Studies Depression (CES-D) scale: screening for depressive symptoms. Sleep was evaluated for the previous week using a four-category scale (never to always). Section of Opiate Treatment Index (OTI) asking patients whether they experienced sleep problems. According to the answers for these questionnaires three groups were identified. No sleep problems, moderate sleep problems, and severe sleep problems were distributed as 39.5, 27.2, and 33.3% at enrolment, 46.0, 34.5, and 19.4% at M6, and 46.5, 28.8, and 26.6% at M12. Patients who reported severe sleep disturbance were significantly more likely to be younger, to have a higher suicidal risk, to experience pain, and to have a higher level of nicotine dependence.
19	Roncero, 2016, Spain	Observational: cross-sectional, descriptive, multicenter, epidemiological study	*N* = 621 (94% were on methadone treatment)	Data extracted from an earlier report of the study (Roncero, 2011): Female: 15.9% Age: 38.89 (7.95) 67% of all evaluated patients presented psychiatric comorbidity (anxiety, mood, sleeping disorders)	Mean MMT dose (mg/day): 61.52 (49.14)	Medical evaluation using DSM-IV-TR criteria. Sleep disorder in 41%, equally distributed across genders and methadone doses.
20	Zahari, 2016, Malaysia	Observational: cross-sectional evaluation	*N* = 165	Female: 0% Age: 37.27 (6.24) years	Mean MMT dose (mg/day): 76.64 mg/day (37.63). Mean MMT duration (years): 2.92 (2.09)	Self-report scale: PSQI: 58.8% of patients reported PSQI > 5.
21	Peles 2017, Israel	Observational: cross-sectional evaluation of sleep quality in patients receiving MMT	*N* = 39 (26 with history of sexual abuse)	Female: 100% Age: 46.9 (9.5) for women who did not have history of abuse, 42.2 (7.6) for women who had history of abuse.	Mean duration in MMT treatment (years): 8.9 (6.6) for women who did not have history of abuse, 7.6 (5.8) for women who had history of abuse.	Self-report scales: PSQI: 38.4% of women without history of abuse and 84.6% women with history of abuse in MMT reported PSQI > 5 ESS: 30.8% of women without history of abuse and 46.1% of women with history of abuse in MMT reported ESS > 7
22	Dunn, 2018, USA	Observational: cross-sectional evaluation	*N* = 125	Female: 52.8% Age 46.1 (10.8) years	Mean MMT duration (years): 5.7 (5.9)	Self-report scale: Medical Outcomes Study (MOS) Sleep Scale Time needed to fall asleep: 0–30 min (47.8%), 31–60 min (28.9%) and > 60 min (23.3%). Participants reported a mean of 5.3 (2.1) hours slept per night. Reported symptoms included non-restorative or restless sleep (20.2%), waking up short of breath or with a headache (19.1%), daytime drowsiness (25.5%), difficulty falling asleep (29.8%), nocturnal awakenings with difficulty returning to sleep (29.8%), difficulty staying awake during the day (26.9%), and snoring (28.0%). These observations were similar to those reported in the buprenorphine group, except for daytime drowsiness. Sleep apnea was reported as baseline medical condition for 12.0% of participants.
23	Li, 2018, Taiwan	Observational: retrospective study exploring the frequency of the newly onset clinically predominant sleep disturbance (CPSD) while in MMT	*N* = 152	Female: 16.4% Age : 42.11 (7.8) years HIV: 5.9% Smokers: 81.6%	Mean MMT dose (mg/day): 69.7 (30)	Time to CPSD (requiring pharmacological or nonpharmacological treatment). 19.1% of the participants had new-onset CPSD. Identified predictors: earlier age at onset of heroin exposure, lower attendance, greater maximum dose of methadone, and shorter time to maximum methadone dose.
24	Hallinan, 2019, Australia	Observational: cross-sectional evaluation	*N* = 184	Female: 51.1% Age 38.9 (8.5) years Heroin consumption: 48.% High risk alcohol use: 25.5% Antipsychotic treatment: 9.2% Antidepressant use: 20.1% Chronic pain: 20.1% Anxiety: 47.8% Depression: 21.2%	Median methadone dose (mg/day): 85 IQR (55–120) Median MMT duration (years): 4.0 IQR (1.5–8.0)	Self-report scales: 27-item Symptoms Checklist: 69.0% reported trouble sleeping, 61.0% reported moderate-to-very important severity of experienced troubles sleeping, 63.4% reported feeling drowsy during the day, 48.9% reported moderate-to-very important severity of feeling drowsy. Athens Insomnia Scale (Athens): 69.6% reported Athens ≥ 6, 55.4% reported Athens ≥ 10. ESS: 22.8% reported ESS ≥ 11, 6.0% reported ESS ≥ 16
25	Le, 2019, Vietnam	Observational: cross-sectional evaluation	*N* = 395	Female: 0 Age 25.9 (7.8) years	Mean MMT duration (years): 2.8 (2.2) Mean methadone dose (mg/day): 69.2 (37.0)	Self-report scale: PSQI: 26.6% reported PSQI > 5. No difference in methadone dosage in patients with or without sleep-related symptoms.
26	Zhong, 2019, China	Observational: cross-sectional evaluation of frequent nightmares in patients receiving MMT	*N* = 603	Female: 30.2% Age 38.1 (7.0) years	Methadone dose: 273 participants with less than 70 mg/day, 330 participants with more than 70 mg/day	Self-report: Frequent nightmares were reported in 25.9% of participants (23.8% in men and 30.4% in women). Frequent nightmares were present in 32.7% of patients with methadone > 70 mg/day and in 18.7% of patients with methadone ≤ 70 mg/day. There was an association with educational level, unemployment, forced labor, injecting heroin before MMT, high dose of methadone, use of hypnotic treatment, presence of HBV, pain, anxiety, and insomnia.
27	Baldassarri, 2020, USA	Observational: cross-sectional evaluation	*N* = 164	Female: 41% Age: 44 (10) years	Mean methadone dose: 80 ± 31 mg Median duration of treatment: 24 months (IQR: 8–48 months)	Self-report scale: PSQI: 90% of participants reported PSQI > 5, the mean PSQI was high (11.0 ± 4). ESS: 46% of participants reported ESS > 10. Higher PSQI: significant association with pain interference, somatization, and negative association with employment. Greater sleepiness: significant association with body mass index.
28	Chinichian and Alemohammad, 2020, Iran	Observational: cross-sectional evaluation	*N* = 382	Female: 0% Age : 34.12 (7.95) years All patients were incarcerated 52.6% used more than one substance Amphetamine use: 22.3% Heroin use: 10.5%	Mean MMT dose (mg/day): 59.95 (23.70)	Self-report scale: PSQI: 87.7% of patients reported PSQI > 5.
29	Elkana, 2020, Israel	Observational: prospective longitudinal study	*N* = 19	Female: 26.3% Age : 51.6 (10.4) years	Mean methadone dose (mg/day): 124.2 (43.4) MMT duration: 1 year	Self-report scale: PSQI: 78.9% reported PSQI > 5
30	Madjova, 2021, Bulgaria	Observational: cross-sectional evaluation	*N* = 81	Female: 23 (28%) Age: 39 (9.07) years Arterial hypertension: 16.1% Obesity: 2.5%	Participants were included if they were in MMT for at least 2 months	Self-report evaluation using investigator-designed survey Sleep problems were reported by 79% (64) of participants, insomnia was reported by 30.9% (25), experience of morning fatigue by 44.4% (36), daytime drowsiness by 56.8% (46), short sleep by 21% (17), snoring by 26% (21), 32% reported problems falling asleep, 79% wake up frequently in the evening. Self-evaluation of sleep apnea in 16.1%.
31	Talaei, 2021, Iran	Observational: cross-sectional evaluation	*N* = 152	Female: 18.8% Age: 42.08 (12.44) years	Participants were enrolled in MMT program	Self-report scales: ESS: 14.5% of participants reported ESS > 11 Fatigue Severity Scale (FSS): 42.5% of participants reported FSS > 36 STOP-BANG test: moderate-to-high risk of apnea was observed in 62.5% of participants. There was a moderate correlation between apnea risk and anxiety.
32	Ellis, 2022, USA	Observational: cross-sectional evaluation in patients with OUD.	*N* = 38	For all patients with OUD (*N* = 154): Female: 39.6% Age: 35.13 (8.42) years	No information	Self-report scale: ISI: 60.5% of participants receiving methadone reported ISI > 15 Insomnia reported in 60.5% of participants
33	Han, 2022, USA	Observational: cross-sectional evaluation	*N* = 47	Female: 23.4% Age: 58.8 (5.8) years 100% of the participants were older than 50 years old Psychiatric comorbidity in 72.4%	Mean methadone dose (mg/day): 95.3 (57.4) Mean MMT duration (months): 81.7 (106.4)	Self-reported sleep-related problems during a 45-minute interview with an investigator. Insomnia was reported by approximately 58% of the participants (estimated from the graph).
34	Maurício, 2022	Observational: cross-sectional evaluation	*N* = 68	Female: 13.2% Age: 51.6 years More than 90% used sleep inducing treatments	Median methadone dose: 39.8 mg/day	Self-report scales: PSQI: 91.2% of participants reported PSQI > 5. There were also complaints about daytime sleepiness.
35	Tsai, 2023, USA	Observational: anonymous survey exploring physical function, pain, sleep, and other health outcomes in patients receiving medications for OUD.	*N* = 40	Female: 20 (50%) Age: 40.95 (12.24) years	66.7% of participants were in Phase one of the program (6 medication visits per week and 3 h of counseling per week)	Self-report evaluation using investigator-designed survey. 50.0% of the participants reported at least moderate sleep problems
36	Guillet, 2024, France	Observational: retrospective cross-sectional study	*N* = 60	Female: 26.7% Age: 36 (7.08) years	Mean MMT dose (mg/day): 81.6 (39.9)	Polygraphy: SAS in 78.3% (95% CI, 66.7% to 88.3%). Moderate to severe SAS in 40%. Severe SAS in 23.3%. Central SAS in 7 patients. ROC-curve exploration: dose cut-off of 77.5 mg/day predicting an AHI ≥ 15 events/h.
37	Nwanaji-Enwerem, 2024, USA	Observational: cross-sectional evaluation of sleep quality in patients receiving MMT, together with a qualitative evaluation	*N* = 120	Female: 40.8% Age: 41.58 (11.64) years	No data on MMT dosage or duration	Self-report scale: ISI: 29% of the participants reported ISI > 15 (clinical insomnia) Reports of higher average pain, stress and psychological distress scores for those with clinically significant insomnia.
38	Sun, 2024, China	Observational: cross-sectional evaluation	*N* = 75, however only 30 still in MMT (other participants with history of MMT)	Reported only for overall sample: Female: 40/75 Age: 3 participants ≤ 40, 20 participants 41–50, 49 participants 51–60, 3 participants > 60 years	Participants were currently or previously enrolled in an MMT program. Only data for currently enrolled patients was extracted.	Self-report scale: PSQI: 23/30 (76.7%) participants reported PSQI > 5

### Data synthesis

The data synthesis method was selected after discussion with the research team. It was decided that studies measuring sleep-related symptoms with a standardized scale were to be pooled in a meta-analysis of prevalence. For this, we used the meta and metafor packages in R using logit transformation, the DerSimonian-Laird estimator for heterogeneity, and inverse-variance weighting [13, 14]. Our results are presented as a forest plot. We planned to explore heterogeneity by performing sensitivity analyses and providing a prediction interval. All other studies were synthesized narratively and in a tabular form.

### Risk of bias and certainty of evidence

Two authors (DP and AD) independently assessed risk of bias at the outcome level using the Checklist for Prevalence Studies from the Joanna Briggs Institute [15]. This assessment covered the target population, study design, sample size, participant characteristics, statistical analysis, outcome measurement methods, and the use of a standard and reliable outcome assessment. The risk-of-bias assessment focused specifically on the prevalence estimates and was not intended to evaluate the overall methodological quality of each study. The overall judgment was based on responses to the signaling questions across all criteria. Studies were considered to have a low risk of bias when all signaling questions were answered “Yes,” a moderate risk of bias when one or two responses were “Unclear,” and a high risk of bias in all other cases.

Potential publication bias was assessed by visual inspection of the funnel plot and through an exploratory Egger’s test.

The certainty of evidence was assessed independently and in duplicate using the GRADE approach [16]. The differences in risk of bias or certainty of evidence evaluations were resolved through discussion. The GRADE approach was adapted for observational evidence, as the research question concerned the prevalence of sleep problems. Observational studies were therefore considered to provide high-certainty evidence for this type of question. The certainty of evidence was subsequently downgraded based on risk of bias, inconsistency, indirectness, and imprecision.

## Results

### General characteristics of the included studies

**Fig. 1:**
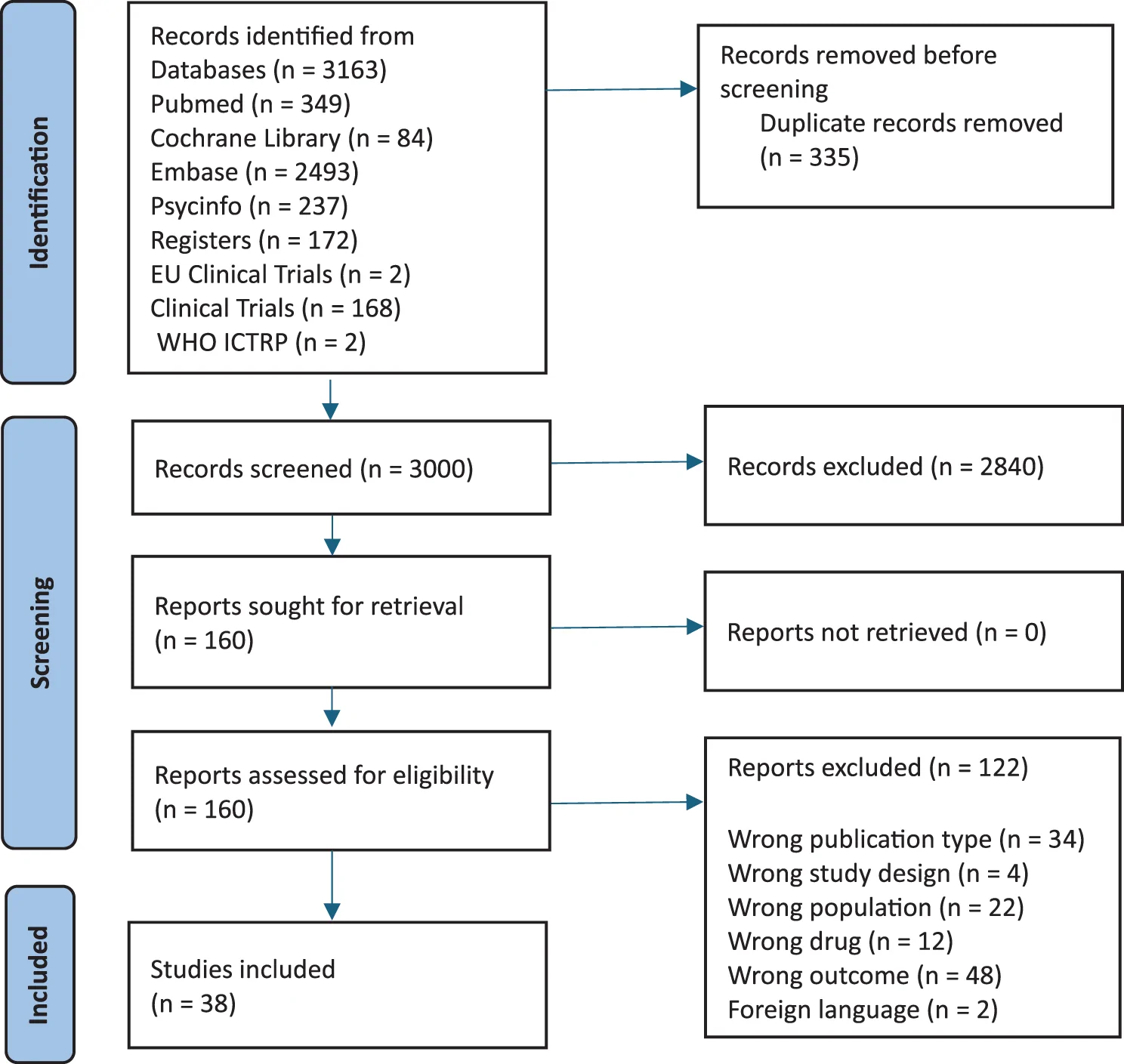
PRISMA flow diagram describing the study selection process

After implementing our search strategy in the various predefined information sources, we obtained 3 335 hits. After semi-automatic duplicate removal, 3 000 articles were screened by two authors in blind mode. The first round of selection based on titles and abstracts resulted in 160 articles left for selection based on full texts using the same selection process (Fig. 1). Finally, 38 articles were included in the systematic review (Table 1). All included articles were published in English between 1981 and 2024, the majority having been published after 2011. Most studies had been conducted in the United States of America [17–25], in European countries [26–32] or in Israel [33–38].

Sample sizes differed considerably. Nineteen studies recruited less than 100 participants, especially in cases where physiological measures were used to characterize sleep-related symptoms [17, 21–23, 25–27, 31, 32, 34, 36–44], and nineteen studies recruited more than 100 participants [18–20, 24, 28–30, 33, 35, 45–54]. In 33 studies, there was a lower proportion of female participants, and in five studies all the participants were male [17, 36, 48, 51, 53]. Only one study included all female participants [37].

Most studies were observational [17–24, 27, 30–44, 46–54], and only five RCTs compared methadone to other treatments [25, 26, 28, 29, 45]. Methadone was dispensed at standard MMT doses, and sleep evaluation was performed after several months or years of treatment. In the included RCTs, sleep-related symptoms were reported as adverse events. In the remaining observational studies, sleep-related symptoms were assessed using standardized scales, physiological measures, and clinical interviews. Among the studies that used validated scales, most [18, 20, 33–35, 37, 38, 41, 43, 44, 46, 48, 51, 53] used the Pittsburgh Sleep Quality Index (PSQI), a validated self-report questionnaire that assesses the quality of sleep over a period of one month [55]. Some studies [20, 35, 37, 40, 41, 50, 54] reported the results from Epworth Sleepiness Scale, a self-report questionnaire allowing participants to assess their daily sleepiness [56]. Physiological measurements were reported using polysomnography and polygraphy techniques [32, 39, 40]. Studies that used clinical evaluation of sleep-related problems had more heterogeneous outcome assessment methods [17, 22, 23, 27, 29–31, 36, 42, 49, 52].

### Meta-analysis of the prevalence of poor sleepers among patients receiving MMT

**Fig. 2:**
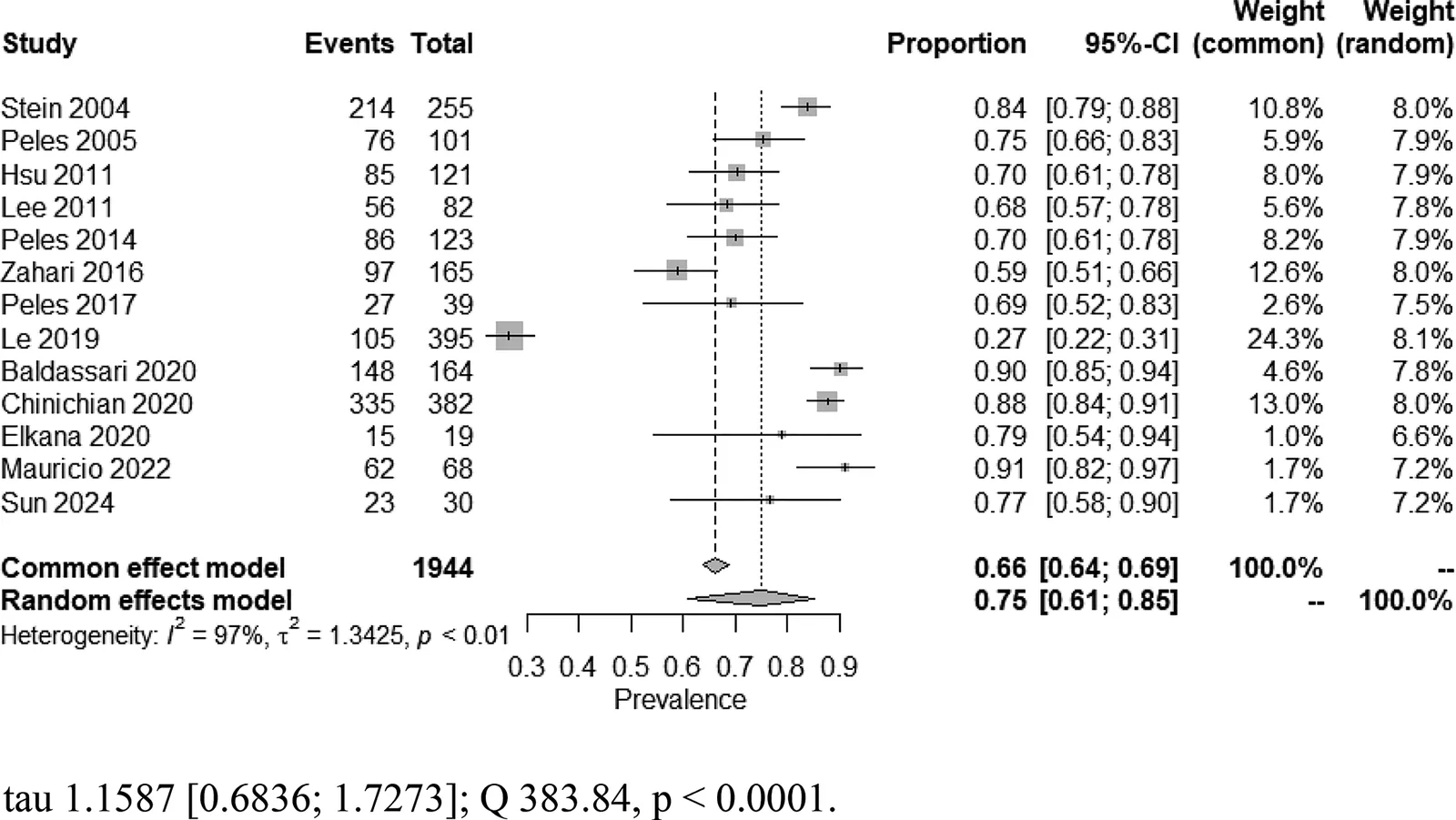
Forest plot of the prevalence of poor sleepers across 13 eligible studies

Thirteen studies explored the prevalence of poor sleepers using a PSQI score > 5 [18, 20, 33, 35, 37, 38, 41, 43, 44, 46, 48, 51, 53]. These studies included 1944 participants, of whom 1329 had sleep problems, defined as a PSQI score > 5. Given the expected heterogeneity across studies, we used a random-effects meta-analysis to obtain a pooled prevalence estimate. Among the included participants receiving MMT, the prevalence of poor sleepers was 75% (95% CI [61–85%], I^2^ 97%). The forest plot presented in Fig. 2 also presents a sensitivity analysis using the common-effects model. We identified one outlier [51].

**Fig. 3:**
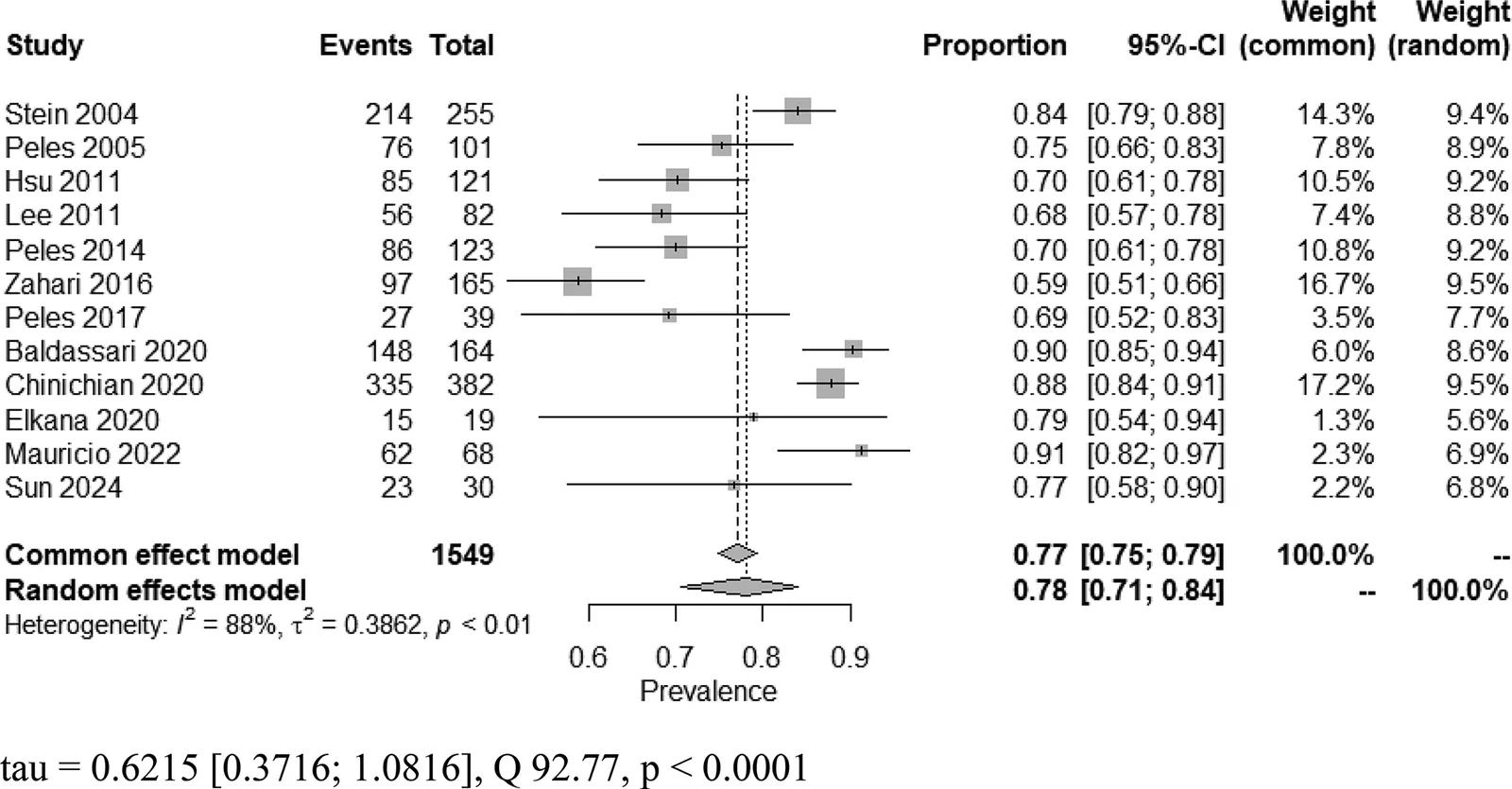
Forest plot of the sensitivity analysis excluding the outlier study by Le et al. 2019

Sensitivity analyses included leave-one-out analyses with removal of the identified outlier, as well as subgroup analyses based on country, mean methadone dose (which may be considered a proxy for OUD severity), and the proportion of female participants. The results of these sensitivity analyses are presented in Figs. 3, and Figures S1-S3 in the Supplement. Overall, the removal of the outlier as well as pooling studies by country, proportion of included women, and methadone dosages reduced the statistical heterogeneity suggesting that country, methadone dosages and proportion of female participants could potentially explain the observed heterogeneity.

Given that some trials investigated sleep-related problems in highly specific populations (e.g., same-sex cohorts or incarcerated participants), we conducted a supplementary analysis excluding the following studies: three including same-sex populations [37, 48, 51]; one including same-sex and incarcerated participants [53]; and another study including only a subsample of patients receiving MMT, with the remaining participants having a history of such treatment [44]. In addition, the Baujat plot (Figure S4 in the Supplement) identified the studies contributing most to the observed heterogeneity [18, 20, 51, 53]. We therefore conducted a sensitivity analysis excluding these studies. These analyses, presented in Figures S5 and S6 in the Supplement, yielded similar effect sizes for the prevalence of poor sleepers, while reducing measures of statistical heterogeneity.

In prevalence meta-analyses, the I² statistic is commonly expected to be high [57]. In accordance with recent recommendations, we addressed this issue by examining the prediction interval, which ranged from 50% to 93%. This finding suggests that, when variability across the included studies is taken into account, the prevalence of poor sleepers in future studies involving participants receiving MMT is likely to range from 50% to 93%. The prediction interval was close to the confidence interval around the pooled estimate, and, in the analysis excluding the outlier study, the τ estimate of absolute heterogeneity was smaller than the pooled prevalence estimate.

**Fig. 4:**
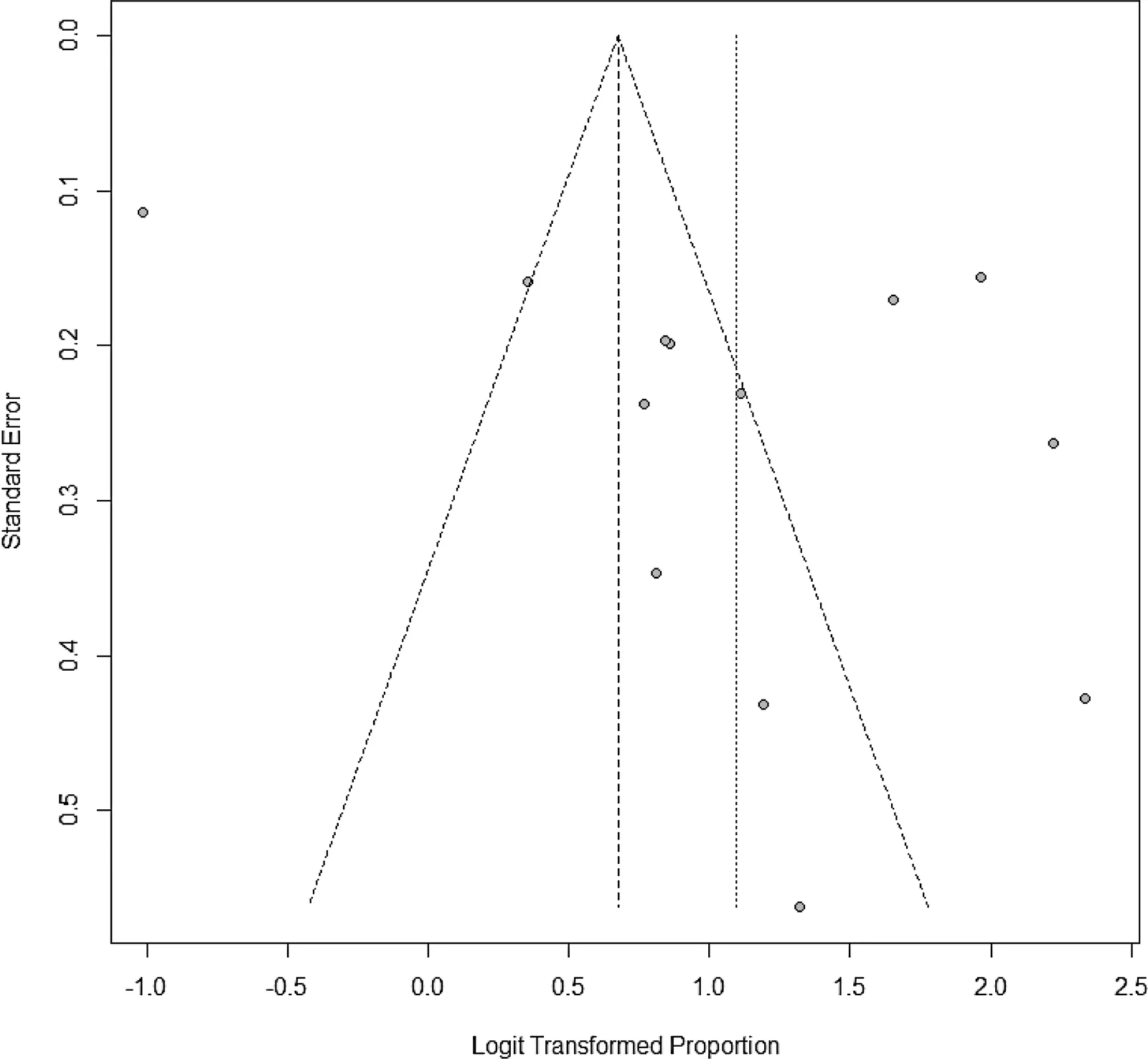
Funnel plot. Exploratory Egger’s test not significant (p = 0.2056)

Of the thirteen trials selected for quantitative synthesis, eight were considered to have a low risk of bias [18, 20, 33, 35, 46, 48, 51, 53], three a high risk of bias [38, 41, 44], and two a moderate risk of bias [37, 43]. The certainty of evidence was judged to be moderate, as there were concerns related to the statistical heterogeneity between studies. Publication bias was not suspected based on visual inspection of the funnel plot (Fig. 4). Egger’s test did not indicate significant small-study effects (*p* = 0.2056). However, given the relatively small number of studies included in the meta-analysis and the associated risk of limited statistical power, and given the test’s limitations for proportion data, the results of the Egger’s test should be interpreted as exploratory. The results of the risk-of-bias assessment and GRADE assessment of the studies pooled for a meta-analysis are presented in Tables 2 and 3.

**Table 2:** Risk-of-bias assessment for the studies included in the meta-analysis

Study ID	1	2	3	4	5	6	7	8	9	Overall risk of bias
Stein, 2004	Y	Y	Y	Y	Y	Y	Y	Y	Y	Low
Peles, 2005	Y	Y	Y	Y	Y	Y	Y	Y	Y	Low
Hsu, 2011	Y	Y	Y	Y	Y	Y	Y	Y	Y	Low
Lee, 2011	Y	U	U	N	Y	Y	Y	Y	Y	High
Peles, 2014	Y	Y	Y	Y	Y	Y	Y	Y	Y	Low
Zahari, 2016	Y	Y	Y	Y	Y	Y	Y	Y	Y	Low
Peles 2017	Y	Y	U	Y	Y	Y	Y	Y	Y	Moderate
Le, 2019	Y	Y	Y	Y	Y	Y	Y	Y	Y	Low
Baldassarri, 2020	Y	Y	Y	Y	Y	Y	Y	Y	Y	Low
Chinichian and Alemohammad, 2020	Y	Y	Y	Y	Y	Y	Y	Y	Y	Low
Elkana, 2020	Y	Y	N	Y	N	Y	Y	Y	N	High
Maurício, 2022	Y	U	U	Y	Y	Y	Y	Y	Y	Moderate
Sun, 2024	N	U	U	N	Y	Y	Y	Y	Y	High

**Table 3:** Adapted GRADE certainty-of-evidence assessment for studies included in the meta-analysis assessing the prevalence of poor sleepers (PSQI>5) **Question**: What is the prevalence of sleep-related problems in MMT patients?

Certainty assessment							Certainty
No of studies	Study design	Risk of bias	Inconsistency	Indirectness	Imprecision	Other considerations	
**prevalence of poor sleepers (assessed with: PSQI > 5)**							
13	Observational evidence (appropriate for the research question)	not serious	serious^a^	not serious	not serious	publication bias not suspected	⨁⨁⨁◯ Moderate^a, b^

**CI**: confidence interval

*Explanations*

a. Moderate heterogeneity

b. Egger’s test not significant

Some studies reported associations between sleep-related symptoms and other factors. Stein et al. reported that there was an association between depression, anxiety, nicotine dependence, pain and unemployment and poor quality of sleep [18]. Baldassari and colleagues discussed the association between PSQI scores and pain, somatization, and unemployment [20]. In Peles et al., PSQI scores were higher in patients with chronic pain, psychiatric comorbidities, and those taking benzodiazepines [33]. Several studies reported the association between PSQI scores and methadone dose [33, 46]. However, a trial by Le et al. found no difference in the methadone doses taken by patients with and without sleep-related problems [51]. There was a higher prevalence of poor sleepers among women with history of sexual abuse [37].

Overall, poor sleep quality was highly prevalent among patients receiving MMT in the included studies. Chronic pain, unemployment, and psychiatric comorbidities were the factors most commonly reported in association with poor sleep quality in individual trials.

### Prevalence of excessive daytime sleepiness and insomnia among patients receiving MMT

Seven studies [20, 35, 37, 40, 41, 50, 54] reported data on daytime sleepiness using ESS. However, because of the considerable heterogeneity in the choice of ESS threshold for daytime sleepiness, a quantitative analysis was not performed. Overall, the prevalence of daytime sleepiness was reported in 15 to 50% of participants, with most trials reporting values between 15 and 25%. Daytime drowsiness was frequently explored as part of clinical interviews. This symptom was reported in 23 to 64% of patients receiving MMT [17, 19, 31, 50]. In the large RCT by Mattick et al., daytime sleepiness was reported by 9% of participants [45]. In the study in which comparisons were made with patients receiving buprenorphine treatment, it was the only symptom that was more prevalent in the methadone group [19].

The Insomnia Severity Index (ISI) [58], a brief self-report screening tool, was used to investigate insomnia in two studies [21, 24]. Insomnia was also assessed through clinical interviews in observational studies [22, 27, 31] and recorded as an adverse event in randomized controlled trials [25, 26, 28, 45]. ISI > 15 was reported in 29% of participants in the study by Nwaraji-Enwerem et al., which also showed that individuals reporting clinical insomnia were those who also reported pain, stress and psychological distress [24]. ISI > 15 was reported in 60.5% of the individuals in a smaller study by Ellis et al. [21]. Complaints about insomnia were reported in 30 to 93% of participants in observational studies using clinical interviews and scales designed by the authors to investigate this symptom [22, 27, 31]. In a large observational study by Wang et al., new-onset insomnia was reported in 18.3% of participants, which contrasts with the previously described observations [47]. Furthermore, in RCTs where sleep-related symptoms were reported as adverse events rather than systematically assessed, insomnia was reported in 1.25% to 20% of participants receiving MMT [25, 26, 28, 45].

### Polysomnography/polygraphy observations in patients receiving MMT

Four studies reported polysomnography or polygraphy observations [32, 34, 39, 40]. In the study by Teichtahl et al., five of ten participants presented sleep-disordered breathing, and six out of ten participants presented central apneas [39]. In a study by Wang et al., 15 of 50 patients presented central apneas [40]. In a study by Guillet et al., the prevalence of sleep apnea syndrome among patients receiving MMT was 78.3%, and severe sleep apnea syndrome was present in 40% [32]. In this study, higher doses of MMT seemed to be associated with greater sleep apnea severity. In the study by Peles et al., sleep apnea syndrome was reported in 39.1% and 43.5% of participants at 6 and 12 months after MMT initiation, respectively [34]. When considering overall sleep architecture, the sleep efficiency of participants receiving MMT was significantly impaired.

### Other sleep-related symptoms

To investigate the prevalence of other sleep-related symptoms in patients receiving MMT, clinical interviews and evaluation scales were used [17, 19, 22, 29, 30, 36, 46, 50, 52]. Some studies based their assessment on author-designed scales [23, 27, 31, 42]. In a large cross-sectional evaluation by Roncero et al. including 621 patients among whom 94% were receiving MMT, sleep disorders, as defined by DSM-IV-TR criteria, were present in 41% of the sample [30]. In the large study by Zhong et al., 25.9% of patients receiving MMT reported frequent nightmares, and prevalence was higher among those receiving higher methadone doses [52]. Reports of frequent nightmares were also associated with educational level, unemployment, forced labor, injected heroin use, use of hypnotics, Hepatitis B, pain, anxiety, and insomnia. In a study by Enguelberg-Gabbay et al., sleep bruxism was diagnosed in 52.2% of patients receiving MMT [36]. In another study, 64.3% of participants reported restless or disturbed sleep, 62.5% reported problems falling asleep, and 53.6% early morning awakenings [42]. In a study conducted by Hallinan et al., 69% of patients reported trouble sleeping [50]. Madjova et al. found that sleep problems were reported by 79% of participants, with 44.4% reporting morning fatigue, 21% short sleep, 26% snoring, 32% problems falling asleep, and 16.1% sleep apnea [31]. Tsai et al. reported that 50% of participants had at least moderate sleep problems [23].

Some studies examined sleep-related problems in relation to MMT from a longitudinal or comparative perspective [19, 29, 49]. In a multisite RCT, Nordmann et al. reported that moderate and severe sleep problems were observed in 34.5% and 19.6% of patients, respectively, after 6 months of follow-up, and in 28.8% and 26.6% after 12 months [29]. These observations should be interpreted in light of the prevalence of moderate and severe sleep problems at enrolment, which was 27.2% and 33.3%, respectively. In a retrospective study by Li et al., new-onset sleep-related problems were reported by 19.1% of patients [49]. When comparison was possible between methadone and buprenorphine maintenance treatment, sleep problems appeared to be distributed similarly across groups, with the exception of daytime drowsiness [19].

### Risk of bias

For the studies not included in the meta-analysis, risk-of-bias assessment is presented in the Appendix (Table S1). Of the 25 studies, 15 were judged to have a low-to-moderate risk of bias [19, 24, 25, 28–30, 32, 34, 36, 40, 45, 47, 49, 50, 52], and ten to have a high risk of bias [17, 21–23, 26, 27, 31, 39, 42, 54]. The risk-of-bias domains most frequently downrated were sample size, participant description, and setting. These concerns reflected the small sample sizes of some studies, the limited information available in abstract-only publications, and insufficient reporting of study settings. There were also concerns about the validity of outcome measurement, as the prevalence of sleep-related problems was sometimes assessed using non-standardized and non-validated tools.

## Discussion

Overall, sleep-related symptoms were highly prevalent among patients receiving MMT in the included studies and were associated with several psychosocial factors. The factors most commonly associated with poor sleep quality were chronic pain, unemployment, and psychiatric comorbidities. Our quantitative synthesis of the trials measuring sleep quality with the PSQI showed that 75% (95% CI [61–85%]) of participants were poor sleepers, and, in future trials, the prevalence of poor sleepers is likely to be between 50% and 93%. A qualitative synthesis of the remaining studies showed that 15 to 50% of participants reported daytime sleepiness, and 30 to 93% of participants reported insomnia. Polysomnography/polygraphy studies showed that participants had reduced sleep efficiency and that 39.1% to 78.3% of participants had sleep apnea syndrome.

Our results are consistent with previous reviews on the prevalence of sleep-related problems among patients receiving MMT. In a review focused on sleep-disordered breathing, Hassamal et al. included six studies conducted in patients receiving MMT [7]. They reported prevalence estimates ranging from 42.3% to 70% for sleep-disordered breathing, from 0% to 60% for central sleep apnea, and from 10% to 35.2% for obstructive sleep apnea. In 2021, Wilkerson and McRae-Clark published a review including 42 studies on sleep in patients receiving medication for OUD, including but not limited to methadone [6]. Their conclusions are consistent with our observations, particularly on the high prevalence of perceived sleep-related problems in patients receiving medications for OUD, and the associations with comorbidities, unemployment, and pain.

From a clinical perspective, several observations should be highlighted. First, although sleep disturbances were highly prevalent among patients receiving MMT, this finding should not be interpreted as evidence that MMT causes these disturbances. Owing to the design of the included studies and the methodological approach used in this review, no causal conclusions can be drawn. Sleep-related problems are frequent among patients with OUD and may reflect not only the pharmacological effects of opioids, but also neuroadaptations associated with OUD, psychiatric comorbidities, and other psychosocial factors [8, 9]. Few studies have explored the dynamic relationship between perceived sleep-related problems and MMT across multiple time points [34, 49]. In the included RCTs, sleep-related problems were reported as adverse events following MMT initiation in 1.25 to 20% of participants [25, 26, 28, 45]. These findings may appear to contrast with cross-sectional evidence suggesting that sleep disturbances often pre-exist MMT. However, sleep-related problems may persist or, in some cases, be exacerbated during MMT, and should therefore be addressed to support recovery.

Second, sleep disturbances seemed to be associated with a range of psychosocial factors, including psychiatric comorbidities, unemployment, and polysubstance use. Patients with OUD frequently present with co-occurring substance use disorders and are often prescribed hypnotic medications, which may complicate the management of sleep disturbances. This underscores the need for a multimodal management approach combining pharmacological and nonpharmacological options. However, well-powered studies investigating pharmacological treatments for sleep-related problems in patients receiving MMT remain limited. Some studies offer preliminary conclusions on the utility of melatonin [59, 60]. Others have explored alternative approaches using herbal medicines [61, 62]. Nonpharmacological interventions have also been investigated, particularly cognitive behavioral therapy and neuromodulation [63, 64]. Finally, clinicians may consider reassessing the indication and utility of hypnotic medications [60], while also providing sleep-related education during patient education sessions, therapy groups, and motivational interventions.

This systematic review has several limitations. First, the included studies were methodologically heterogeneous. However, this heterogeneity partly reflects our a priori decision to include evidence from cross-sectional studies, cohort studies, and adverse event reports from RCTs. Second, some reports were available only as conference abstracts or abstracts published in scientific journals. We chose to include these reports to capture grey literature and reduce the risk of missing relevant data. Third, some included studies were judged to have a high risk of bias, which contributed to downgrading the certainty of evidence. However, the risk-of-bias assessment was performed conservatively: one “Unclear” response to a signaling question led to downgrading, and three “Unclear” responses were sufficient to classify a study as being at high risk of bias.

Our systematic review also has several strengths. To the best of our knowledge, this is the first systematic review and meta-analysis to estimate the prevalence of sleep-related disturbances in patients receiving MMT, using a quantitative approach for studies that reported the prevalence of poor sleepers. Our analysis also incorporated sensitivity analyses to examine additional hypotheses on factors contributing to a higher prevalence of sleep problems. Our risk of bias and certainty of evidence assessment for the trials included in the meta-analysis allowed us to rate the certainty of evidence as moderate. However, important uncertainties remain, as the outlier study identified in our analyses was the largest in terms of sample size [51]. Therefore, additional large studies are needed to obtain a more precise estimate of the prevalence of poor sleepers in populations receiving MMT.

Our conclusions have important clinical implications. In particular, the systematic assessment of sleep-related disturbances in patients with OUD, whether receiving MMT or not, may be clinically valuable when conducted using standardized and validated instruments. Special attention should be paid to sleep disturbances prior to treatment initiation. Clinicians should consider offering multimodal interventions for patients presenting with sleep-related difficulties, taking into account psychiatric comorbidities, polysubstance use, and socioeconomic factors such as unemployment, and they should carefully weigh the indication for hypnotic medications in this population.

## Supplementary Information

Below is the link to the electronic supplementary material.

Supplementary Material 1

## Data Availability

No datasets were generated or analysed during the current study.

## Abbreviations

MMT: Methadone maintenance treatment
OUD: Opioid use disorder
RCT: Randomized controlled trial
PSQI: Pittsburgh Sleep Quality Index
CI: Confidence interval
ESS: Epworth Sleepiness Scale
DSM-IV-TR: Diagnostic and Statistical Manual of Mental Disorders, Fourth Edition, text revision
