# Supplementary material for: Prevalence of sleep-related symptoms in patients receiving methadone maintenance treatment: a systematic review and meta-analysis
Source: Addict Sci Clin Pract. 2026 Jun 19;21:65. doi: 10.1186/s13722-026-00690-2 (PMC13556095; doi:10.1186/s13722-026-00690-2)
Supplement: Supplementary file 1 [file 13722_2026_690_MOESM1_ESM.docx]

**Prevalence of Sleep-Related Symptoms in Patients Undergoing Methadone Maintenance Treatment: A Systematic Review and Meta-Analysis**

Current Addiction Reports

Domitille PALIX^a^, Benjamin PETIT^a^, Benoit TROJAK^a,b^, Anastasia DEMINA*^a,b^

a Addiction Medicine Department, Dijon Bourgogne University Hospital, Dijon, France

b INSERM U1093, CAPS, Université de Bourgogne, UFR STAPS, Dijon, France

* Corresponding author: anastasia.demina@chu-dijon.fr

Document S1: Search strategy

**Pubmed**

((insomnia [Mesh Terms] OR sleep initiation and maintenance disorders [Mesh Terms] OR sleep disorder OR sleep wake disorder [Mesh Terms] OR sleep-related breathing disorders OR sleep apnea OR sleep apnea syndromes [Mesh Terms] OR sleep apnea central [Mesh Terms] OR sleep apnea obstructive [Mesh Terms] OR sleep-related hypoventilation syndromes OR sleep-related hypoxemic syndromes OR other sleep-related breathing disorder OR hypersomnia OR disorders of excessive somnolence [Mesh Terms] OR idiopathic hypersomnia [Mesh Terms] OR sleep apnea syndromes [Mesh Terms] OR narcolepsy [Mesh Terms] OR circadian rhythm sleep disorder OR parasomnia [Mesh Terms] OR REM sleep parasomnias [Mesh Terms] OR disorders of arousal OR sleep arousal disorders [Mesh Terms] OR confusional arousals OR sleepwalking OR somnambulism [Mesh Terms] OR sleep terrors OR night terrors [Mesh Terms] OR sleep paralysis [Mesh Terms] OR nightmare disorder OR sleep-related dissociative disorders OR sleep enuresis OR nocturnal enuresis [Mesh Terms] OR sleep-related groaning OR sleep bruxism OR sleep-related catathrenia OR sleep-related movement disorders OR restless legs syndrome [Mesh Terms] OR periodic limb movement sleep disorder OR sleep-related leg cramps OR sleep-related bruxism OR sleep-related rhythmic movement disorder OR sleep-related movement disorder unspecified OR long sleeper OR short sleeper OR snoring [Mesh Terms] OR sleep talking OR sleep-wake transitions disorders [Mesh Terms] OR disorders of initiating and maintaining sleep OR sleeplessness OR early awakening OR sleep initiation dysfunction OR sleep deprivation [Mesh Terms] OR nocturnal myoclonus syndrome [Mesh Terms] OR nocturnal paroxysmal dystonia [Mesh Terms] OR sleep arousal disorders OR sleep-wake transition disorders OR intrinsic sleep disorder OR sleep state misperception OR sleep-wake schedule disorders OR sleep-wake cycle disorder OR disturbed nyctohemeral rhythm OR advanced sleep phase syndrome OR delayed sleep phase syndrome OR dyssomnias [Mesh Terms] OR extrinsic sleep disorder OR disorders of excessive somnolence OR sleep disorders circadian rhythm [Mesh Terms] OR sleep disorders intrinsic [Mesh Terms] OR restless sleep disorder [Mesh Terms])) AND ((opioid maintenance treatment OR opiate maintenance treatment OR opioid substitution treatment OR opiate substitution treatment [Mesh Terms] OR methadone [Mesh Terms] OR D-methadone [Mesh Terms] OR 6S-methadone OR 6S-6-dimethylamino-4,4 diphenyl-3-heptanone OR 6-dimethylamino-4,4-diphenylhexan-3-one OR phenadone OR physeptone OR phymet OR dolophine OR amidone OR biodone OR metadol-d OR metasedin OR methaddict OR methadose OR methex OR pinadone OR symoron))

**EMBASE**

(‘insomnia’ OR ‘sleep initiation and maintenance disorders’ OR ‘sleep disorder’ OR ‘sleep wake disorder’ OR ‘sleep-related breathing disorders’ OR ‘sleep apnea’ OR ‘sleep apnea syndromes’ OR ‘sleep apnea central’ OR ‘sleep apnea obstructive’ OR ‘sleep-related hypoventilation syndromes’ OR ‘sleep-related hypoxemic syndromes’ OR ‘other sleep-related breathing disorder’ OR ‘hypersomnia’ OR ‘disorders of excessive somnolence’ OR ‘idiopathic hypersomnia’ OR ‘sleep apnea syndromes’ OR ‘narcolepsy’ OR ‘circadian rhythm sleep disorder’ OR ‘parasomnia’ OR ‘REM sleep parasomnias’ OR ‘disorders of arousal’ OR ‘sleep arousal disorders’ OR ‘confusional arousals’ OR ‘sleepwalking’ OR ‘somnambulism’ OR ‘sleep terrors’ OR ‘night terrors’ OR ‘sleep paralysis’ OR ‘nightmare disorder’ OR ‘sleep-related dissociative disorders’ OR ‘sleep enuresis’ OR ‘nocturnal enuresis’ OR ‘sleep-related groaning’ OR ‘sleep bruxism’ OR ‘sleep-related catathrenia’ OR ‘sleep-related movement disorders’ OR ‘restless legs syndrome’ OR ‘periodic limb movement sleep disorder’ OR ‘sleep-related leg cramps’ OR ‘sleep-related bruxism’ OR ‘sleep-related rhythmic movement disorder’ OR ‘sleep-related movement disorder unspecified’ OR ‘long sleeper’ OR ‘short sleeper’ OR ‘snoring’ OR ‘sleep talking’ OR ‘sleep-wake transitions disorders’ OR ‘disorders of initiating and maintaining sleep’ OR ‘sleeplessness’ OR ‘early awakening’ OR ‘sleep initiation dysfunction’ OR ‘sleep deprivation’ OR ‘nocturnal myoclonus syndrome’ OR ‘nocturnal paroxysmal dystonia’ OR ‘sleep arousal disorders’ OR ‘sleep-wake transition disorders’ OR ‘intrinsic sleep disorder’ OR ‘sleep state misperception’ OR ‘sleep-wake schedule disorders’ OR ‘sleep-wake cycle disorder’ OR ‘disturbed nyctohemeral rhythm’ OR ‘advanced sleep phase syndrome’ OR ‘delayed sleep phase syndrome’ OR ‘dyssomnias’ OR ‘extrinsic sleep disorder’ OR ‘disorders of excessive somnolence’ OR ‘sleep disorders circadian rhythm’ OR ‘sleep disorders intrinsic’ OR ‘restless sleep disorder’) AND (‘opioid maintenance treatment’ OR ‘opiate maintenance treatment’ OR ‘opioid substitution treatment’ OR ‘opiate substitution treatment’ OR ‘methadone’ OR ‘D-methadone’ OR ‘6S-methadone’ OR ‘6S-6-dimethylamino-4,4 diphenyl-3-heptanone’ OR ‘6-dimethylamino-4,4-diphenylhexan-3-one’ OR ‘phenadone’ OR ‘physeptone’ OR ‘phymet’ OR ‘dolophine’ OR ‘amidone’ OR ‘biodone’ OR ‘metadol-d’ OR ‘metasedin’ OR ‘methaddict’ OR ‘methadose’ OR ‘methex’ OR ‘pinadone’ OR ‘symoron’)

**Psychinfo**

((insomnia) OR (sleep initiation and maintenance disorders) OR (sleep disorder) OR (sleep wake disorder) OR (sleep-related breathing disorders) OR (sleep apnea) OR (sleep apnea syndromes) OR (sleep apnea central) OR (sleep apnea obstructive) OR (sleep-related hypoventilation syndromes) OR (sleep-related hypoxemic syndromes) OR (other sleep-related breathing disorder) OR (hypersomnia) OR (disorders of excessive somnolence) OR (idiopathic hypersomnia) OR (sleep apnea syndromes) OR (narcolepsy) OR (circadian rhythm sleep disorder) OR (parasomnia) OR (REM sleep parasomnias) OR (disorders of arousal) OR (sleep arousal disorders) OR (confusional arousals) OR (sleepwalking) OR (somnambulism) OR (sleep terrors) OR (night terrors) OR (sleep paralysis) OR (nightmare disorder) OR (sleep-related dissociative disorders) OR (sleep enuresis) OR (nocturnal enuresis) OR (sleep-related groaning) OR (sleep bruxism) OR (sleep-related catathrenia) OR (sleep-related movement disorders) OR (restless legs syndrome) OR (periodic limb movement sleep disorder) OR (sleep-related leg cramps) OR (sleep-related bruxism) OR (sleep-related rhythmic movement disorder) OR (sleep-related movement disorder unspecified) OR (long sleeper) OR (short sleeper) OR (snoring) OR (sleep talking) OR (sleep-wake transitions disorders) OR (disorders of initiating and maintaining sleep) OR (sleeplessness) OR (early awakening) OR (sleep initiation dysfunction) OR (sleep deprivation) OR (nocturnal myoclonus syndrome) OR (nocturnal paroxysmal dystonia) OR (sleep arousal disorders) OR (sleep-wake transition disorders) OR (intrinsic sleep disorder) OR (sleep state misperception) OR (sleep-wake schedule disorders) OR (sleep-wake cycle disorder) OR (disturbed nyctohemeral rhythm) OR (advanced sleep phase syndrome) OR (delayed sleep phase syndrome) OR (dyssomnias) OR (extrinsic sleep disorder) OR (disorders of excessive somnolence) OR (sleep disorders circadian rhythm) OR (sleep disorders intrinsic) OR (restless sleep disorder)) AND ((opioid maintenance treatment) OR (opiate maintenance treatment) OR (opioid substitution treatment) OR (opiate substitution treatment) OR (methadone) OR (D-methadone) OR (6S-methadone) OR (6S-6-dimethylamino-4,4 diphenyl-3-heptanone) OR (6-dimethylamino-4,4-diphenylhexan-3-one) OR (phenadone) OR (physeptone) OR (phymet) OR (dolophine) OR (amidone) OR (biodone) OR (metadol-d) OR (metasedin) OR (methaddict) OR (methadose) OR (methex) OR (pinadone) OR (symoron))

**Cochrane Library**

#1	MeSH descriptor: [Sleep Initiation and Maintenance Disorders] explode all trees
#2	sleep disorder
#3	MeSH descriptor: [Sleep Wake Disorders] explode all trees
#4	sleep-related breathing disorders
#5	sleep apnea
#6	MeSH descriptor: [Sleep Apnea Syndromes] explode all trees
#7	MeSH descriptor: [Sleep Apnea, Central] explode all trees
#8	MeSH descriptor: [Sleep Apnea, Obstructive] explode all trees
#9	sleep-related hypoventilation syndromes
#10	sleep-related hypoxemic syndromes
#11	other sleep-related breathing disorder
#12	hypersomnia
#13	MeSH descriptor: [Disorders of Excessive Somnolence] explode all trees
#14	MeSH descriptor: [Idiopathic Hypersomnia] explode all trees
#15	MeSH descriptor: [Sleep Apnea Syndromes] explode all trees
#16	MeSH descriptor: [Narcolepsy] explode all trees
#17	circadian rhythm sleep disorder
#18	MeSH descriptor: [Parasomnias] explode all trees
#19	MeSH descriptor: [REM Sleep Parasomnias] explode all trees
#20	disorders of arousal
#21	MeSH descriptor: [Sleep Arousal Disorders] explode all trees
#22	confusional arousals
#23	sleepwalking
#24	MeSH descriptor: [Somnambulism] explode all trees
#25	sleep terrors
#26	MeSH descriptor: [Night Terrors] explode all trees
#27	MeSH descriptor: [Sleep Paralysis] explode all trees
#28	nightmare disorder
#29	sleep-related dissociative disorders

#30	sleep enuresis
#31	MeSH descriptor: [Nocturnal Enuresis] explode all trees
#32	sleep related groaning
#33	sleep bruxism
#34	sleep-related movement disorders
#35	MeSH descriptor: [Restless Legs Syndrome] explode all trees
#36	periodic limb movement sleep disorder
#37	sleep-related leg cramps
#38	sleep-related bruxism
#39	sleep-related rhythmic movement disorder
#40	sleep-related movement disorder unspecified
#41	long sleeper
#42	short sleeper
#43	MeSH descriptor: [Snoring] explode all trees
#44	sleep talking
#45	MeSH descriptor: [Sleep-Wake Transition Disorders] explode all trees
#46	disorders of initiating and maintaining sleep
#47	sleeplessness
#48	early awakening
#49	sleep initiation dysfunction
#50	MeSH descriptor: [Sleep Deprivation] explode all trees
#51	MeSH descriptor: [Nocturnal Myoclonus Syndrome] explode all trees
#52	MeSH descriptor: [Nocturnal Paroxysmal Dystonia] explode all trees
#53	sleep arousal disorders
#54	sleep-wake transition disorders
#55	intrinsic sleep disorder
#56	sleep state misperception
#57	sleep-wake schedule disorders
#58	sleep-wake cycle disorder
#59	advanced sleep phase syndrome
#60	delayed sleep phase syndrome
#61	MeSH descriptor: [Dyssomnias] explode all trees
#62	extrinsic sleep disorder
#63	disorders of excessive somnolence
#64	MeSH descriptor: [Sleep Disorders, Circadian Rhythm] explode all trees
#65	MeSH descriptor: [Sleep Disorders, Intrinsic] explode all trees
#66	restless sleep disorder
#67	opioid maintenance treatment
#68	opiate maintenance treatment
#69	opioid substitution treatment
#70	MeSH descriptor: [Opiate Substitution Treatment] explode all trees
#71	MeSH descriptor: [Methadone] explode all trees
#72	D-methadone
#73	6S methadone
#74	phenadone
#75	physeptone
#76	phymet
#77	dolophine
#78	amidone
#79	biodone
#80	metasedin
#81	methaddict

#82	methadose
#83	methex
#84	pinadone
#85	symoron

#86	#1 OR #2 OR #3 OR #4 OR #5 OR #6 OR #7 OR #8 OR #9 OR #10 OR #11 OR #12 OR #13 OR #14 OR #15 OR #16 OR #17 OR #18 OR #19 OR #20 OR #21 OR #22 OR #23 OR #24 OR #25 OR #26 OR #27 OR #28 OR #29 OR #30 OR #31 OR #32 OR #33 OR #34 OR #35 OR #36 OR #37 OR #38 OR #39 OR #40 OR #41 OR #42 OR #43 OR #44 OR #45 OR #46 OR #47 OR #48 OR #49 OR #50 OR #51 OR #52 OR #53 OR #54 OR #55 OR #56 OR #57 OR #58 OR #59 OR #60 OR #61 OR #62 OR #63 OR #64 OR #65 OR #66

#87	#67 OR #68 OR # # # # # # OR #70 OR #71 OR #72 OR #73 OR #74 OR #75 OR #76 OR #77 OR #78 OR #79 OR #80 OR #81 OR #82 OR #83 OR #84 OR #85

#88	#86 AND #87

**WHO ICTRP**

(“insomnia” OR “sleep initiation and maintenance disorders” OR “sleep disorder” OR “sleep wake disorder” OR “sleep-related breathing disorders” OR “sleep apnea” OR “sleep apnea syndromes” OR “sleep apnea central” OR “sleep apnea obstructive” OR “sleep-related hypoventilation syndromes” OR “sleep-related hypoxemic syndromes” OR “other sleep-related breathing disorder” OR “hypersomnia” OR “disorders of excessive somnolence” OR “idiopathic hypersomnia” OR “sleep apnea syndromes” OR “narcolepsy” OR “circadian rhythm sleep disorder” OR “parasomnia” OR “REM sleep parasomnias” OR “disorders of arousal” OR “sleep arousal disorders” OR “confusional arousals” OR “sleepwalking” OR “somnambulism” OR “sleep terrors” OR “night terrors” OR “sleep paralysis” OR “nightmare disorder” OR “sleep-related dissociative disorders” OR “sleep enuresis” OR “nocturnal enuresis” OR “sleep-related groaning” OR “sleep bruxism” OR “sleep-related catathrenia” OR “sleep-related movement disorders” OR “restless legs syndrome” OR “periodic limb movement sleep disorder” OR “sleep-related leg cramps” OR “sleep-related bruxism” OR “sleep-related rhythmic movement disorder” OR “sleep-related movement disorder unspecified” OR “long sleeper” OR “short sleeper” OR “snoring” OR “sleep talking” OR “sleep-wake transitions disorders” OR “disorders of initiating and maintaining sleep” OR “sleeplessness” OR “early awakening” OR “sleep initiation dysfunction” OR “sleep deprivation” OR “nocturnal myoclonus syndrome” OR “nocturnal paroxysmal dystonia” OR “sleep arousal disorders” OR “sleep-wake transition disorders” OR “intrinsic sleep disorder” OR “sleep state misperception” OR “sleep-wake schedule disorders” OR “sleep-wake cycle disorder” OR “disturbed nyctohemeral rhythm” OR “advanced sleep phase syndrome” OR “delayed sleep phase syndrome” OR “dyssomnias” OR “extrinsic sleep disorder” OR “disorders of excessive somnolence” OR “sleep disorders circadian rhythm OR “sleep disorders intrinsic” OR “restless sleep disorder”) AND (“opioid maintenance treatment” OR “opiate maintenance treatment” OR “opioid substitution treatment” OR “opiate substitution treatment” OR “methadone” OR “D-methadone” OR “6S-methadone” OR “6S-6-dimethylamino-4,4 diphenyl-3-heptanone” OR “6-dimethylamino-4,4-diphenylhexan-3-one” OR “phenadone” OR “physeptone” OR “phymet” OR “dolophine” OR “amidone” OR “biodone” OR “metadol-d” OR “metasedin” OR “methaddict” OR “methadose” OR “methex” OR “pinadone” OR “symoron”)

**Clinicaltrials.gov**

((insomnia) OR (sleep initiation and maintenance disorders) OR (sleep disorder) OR (sleep wake disorder) OR (sleep-related breathing disorders) OR (sleep apnea) OR (sleep apnea syndromes) OR (sleep apnea central) OR (sleep apnea obstructive) OR (sleep-related hypoventilation syndromes) OR (sleep-related hypoxemic syndromes) OR (other sleep-related breathing disorder) OR (hypersomnia) OR (disorders of excessive somnolence) OR (idiopathic hypersomnia) OR (sleep apnea syndromes) OR (narcolepsy) OR (circadian rhythm sleep disorder) OR (parasomnia) OR (REM sleep parasomnias) OR (disorders of arousal) OR (sleep arousal disorders) OR (confusional arousals) OR (sleepwalking) OR (somnambulism) OR (sleep terrors) OR (night terrors) OR (sleep paralysis) OR (nightmare disorder) OR (sleep-related dissociative disorders) OR (sleep enuresis) OR (nocturnal enuresis) OR (sleep-related groaning) OR (sleep bruxism) OR (sleep-related catathrenia) OR (sleep-related movement disorders) OR (restless legs syndrome) OR (periodic limb movement sleep disorder) OR (sleep-related leg cramps) OR (sleep-related bruxism) OR (sleep-related rhythmic movement disorder) OR (sleep-related movement disorder unspecified) OR (long sleeper) OR (short sleeper) OR (snoring) OR (sleep talking) OR (sleep-wake transitions disorders) OR (disorders of initiating and maintaining sleep) OR (sleeplessness) OR (early awakening) OR (sleep initiation dysfunction) OR (sleep deprivation) OR (nocturnal myoclonus syndrome) OR (nocturnal paroxysmal dystonia) OR (sleep arousal disorders) OR (sleep-wake transition disorders) OR (intrinsic sleep disorder) OR (sleep state misperception) OR (sleep-wake schedule disorders) OR (sleep-wake cycle disorder) OR (disturbed nyctohemeral rhythm) OR (advanced sleep phase syndrome) OR (delayed sleep phase syndrome) OR (dyssomnias) OR (extrinsic sleep disorder) OR (disorders of excessive somnolence) OR (sleep disorders circadian rhythm) OR (sleep disorders intrinsic) OR (restless sleep disorder)) AND ((opioid maintenance treatment) OR (opiate maintenance treatment) OR (opioid substitution treatment) OR (opiate substitution treatment) OR (methadone) OR (D-methadone) OR (6S-methadone) OR (6S-6-dimethylamino-4,4 diphenyl-3-heptanone) OR (6-dimethylamino-4,4-diphenylhexan-3-one) OR (phenadone) OR (physeptone) OR (phymet) OR (dolophine) OR (amidone) OR (biodone) OR (metadol-d) OR (metasedin) OR (methaddict) OR (methadose) OR (methex) OR (pinadone) OR (symoron))

**EU Clinical trials**

((insomnia) OR (sleep initiation and maintenance disorders) OR (sleep disorder) OR (sleep wake disorder) OR (sleep-related breathing disorders) OR (sleep apnea) OR (sleep apnea syndromes) OR (sleep apnea central) OR (sleep apnea obstructive) OR (sleep-related hypoventilation syndromes) OR (sleep-related hypoxemic syndromes) OR (other sleep-related breathing disorder) OR (hypersomnia) OR (disorders of excessive somnolence) OR (idiopathic hypersomnia) OR (sleep apnea syndromes) OR (narcolepsy) OR (circadian rhythm sleep disorder) OR (parasomnia) OR (REM sleep parasomnias) OR (disorders of arousal) OR (sleep arousal disorders) OR (confusional arousals) OR (sleepwalking) OR (somnambulism) OR (sleep terrors) OR (night terrors) OR (sleep paralysis) OR (nightmare disorder) OR (sleep-related dissociative disorders) OR (sleep enuresis) OR (nocturnal enuresis) OR (sleep-related groaning) OR (sleep bruxism) OR (sleep-related catathrenia) OR (sleep-related movement disorders) OR (restless legs syndrome) OR (periodic limb movement sleep disorder) OR (sleep-related leg cramps) OR (sleep-related bruxism) OR (sleep-related rhythmic movement disorder) OR (sleep-related movement disorder unspecified) OR (long sleeper) OR (short sleeper) OR (snoring) OR (sleep talking) OR (sleep-wake transitions disorders) OR (disorders of initiating and maintaining sleep) OR (sleeplessness) OR (early awakening) OR (sleep initiation dysfunction) OR (sleep deprivation) OR (nocturnal myoclonus syndrome) OR (nocturnal paroxysmal dystonia) OR (sleep arousal disorders) OR (sleep-wake transition disorders) OR (intrinsic sleep disorder) OR (sleep state misperception) OR (sleep-wake schedule disorders) OR (sleep-wake cycle disorder) OR (disturbed nyctohemeral rhythm) OR (advanced sleep phase syndrome) OR (delayed sleep phase syndrome) OR (dyssomnias) OR (extrinsic sleep disorder) OR (disorders of excessive somnolence) OR (sleep disorders circadian rhythm) OR (sleep disorders intrinsic) OR (restless sleep disorder)) AND ((opioid maintenance treatment) OR (opiate maintenance treatment) OR (opioid substitution treatment) OR (opiate substitution treatment) OR (methadone) OR (D-methadone) OR (6S-methadone) OR (6S-6-dimethylamino-4,4 diphenyl-3-heptanone) OR (6-dimethylamino-4,4-diphenylhexan-3-one) OR (phenadone) OR (physeptone) OR (phymet) OR (dolophine) OR (amidone) OR (biodone) OR (metadol-d) OR (metasedin) OR (methaddict) OR (methadose) OR (methex) OR (pinadone) OR (symoron))

Figure S1: Subgroup analysis based on the country of recruitment

Figure S2: Subgroup analysis based on mean methadone dose, categorized as <100 mg or ≥100 mg.

Figure S3: Subgroup analysis based on the proportion of female participants, categorized as <30% or ≥30%

Figure S4: Baujat plot identifying studies contributing to the observed statistical heterogeneity

tau = 0.5442 [0.2936; 1.2280], Q 38.34, p < 0.0001

Figure S5: Sensitivity analysis excluding studies in highly specific populations

tau = 0.3799 [0.1599; 0.9912], Q 24.43, p = 0.0019

Figure S6: Sensitivity analysis excluding studies based on Baujat plot

Table S1: Risk of bias evaluation for the studies not included in the quantitative synthesis

| **Study ID** | **1** | **2** | **3** | **4** | **5** | **6** | **7** | **8** | **9** | **Overall risk of bias** |
| --- | --- | --- | --- | --- | --- | --- | --- | --- | --- | --- |
| Langrod, 1981 | Y | Y | Y | N | Y | N | U | U | Y | High |
| Teichtahl, 2001 | Y | U | N | Y | Y | U | U | U | Y | High |
| Mattick, 2002 | Y | Y | Y | Y | Y | U | U | Y | Y | Moderate |
| Lofwall, 2005 | Y | Y | U | Y | Y | U | U | Y | Y | Moderate |
| Wang, 2008 | Y | Y | U | Y | Y | Y | Y | Y | Y | Moderate |
| Wolstein, 2009 | Y | Y | U | Y | Y | U | U | Y | Y | High |
| Peles, 2011 | Y | Y | U | Y | Y | Y | Y | Y | Y | Moderate |
| Wang, 2012 | Y | Y | Y | Y | Y | Y | Y | Y | Y | Low |
| Nkire, 2013 | Y | U | U | U | U | N | U | Y | U | High |
| Cernovsky, 2014 | Y | Y | U | U | Y | N | U | Y | Y | High |
| Hämmig, 2014 | Y | Y | Y | Y | Y | U | Y | U | Y | Moderate |
| Enguelberg-Gabbay, 2016 | Y | Y | U | Y | Y | Y | Y | Y | Y | Moderate |
| Nordmann, 2016 | Y | Y | Y | Y | Y | U | Y | U | Y | Moderate |
| Roncero, 2016 | Y | Y | Y | Y | Y | Y | Y | Y | Y | Low |
| Dunn, 2018 | Y | Y | Y | Y | Y | Y | Y | Y | Y | Low |
| Li, 2018 | Y | Y | Y | Y | Y | U | U | Y | Y | Moderate |
| Hallinan, 2019 | Y | Y | Y | Y | Y | Y | Y | Y | Y | Low |
| Zhong, 2019 | Y | Y | Y | Y | Y | U | U | Y | Y | Moderate |
| Madjova, 2021 | Y | Y | U | U | Y | N | Y | U | Y | High |
| Talaei, 2021 | Y | Y | Y | N | Y | Y | Y | Y | Y | High |
| Ellis, 2022 | U | Y | U | N | Y | Y | Y | Y | Y | High |
| Han, 2022 | Y | Y | U | Y | Y | U | U | Y | Y | High |
| Tsai, 2023 | Y | Y | U | Y | Y | N | U | Y | Y | High |
| Guillet, 2024 | Y | Y | U | Y | Y | Y | Y | Y | Y | Moderate |
| Nwanaji-Enwerem, 2024 | Y | Y | Y | Y | Y | Y | Y | Y | Y | Low |
